# Social Support and Loneliness Among Chinese Caregivers of Children With Chronic Kidney Disease During the COVID-19 Pandemic: A Propensity Score Matching Analysis

**DOI:** 10.3389/fped.2020.570535

**Published:** 2021-01-15

**Authors:** Lin Shi, Hengci Zhang, Haiping Yang, Daoqi Wu, Xiaoqin Li, Yunzhi Zhang, Xuelan Chen, Mo Wang

**Affiliations:** ^1^Department of Nephrology, Children's Hospital of Chongqing Medical University, National Clinical Research Center for Child Health and Disorders, Ministry of Education Key Laboratory of Child Development and Disorders, Chongqing Key Laboratory of Pediatrics, Chongqing, China; ^2^Department of Pediatrics, The Affiliated Hospital of Guizhou Medical University, Guiyang, China; ^3^Department of Infectious Diseases of The Second Affiliated Hospital of Chongqing Medical University, Chongqing, China

**Keywords:** COVID-19, chronic kidney disease, caregivers, propensity score matching, social support, loneliness

## Abstract

To evaluate social support and loneliness as well as their association among caregivers of children with chronic kidney disease (CKD) from China during the coronavirus disease 2019 (COVID-19) pandemic. We collected data for caregivers of children with CKD and caregivers of healthy children and matched the two groups using propensity score matching (PSM). We compared the differences in social support and loneliness between the two groups after matching and analyzed the relationship between social support and loneliness in the observation group. Before PSM, we analyzed the data for 247 caregivers of children with CKD and 315 caregivers of healthy children from 13 provinces. After PSM, the two groups each included 202 caregivers. The social support score of caregivers of children with CKD was lower than that of caregivers of healthy children (*P* < 0.002), while the loneliness score was higher for caregivers of children with CKD than for caregivers of healthy children (*P* < 0.008). The social support score was negatively correlated with the loneliness score (*r* = −0.598, *P* < 0.001). Caregivers of children with CKD experienced less social support and greater loneliness than caregivers of healthy children during the COVID-19 pandemic. Therefore, greater attention should be paid to providing social support for caregivers of CKD children and to improving the ability of these caregivers to cope with loneliness.

## Introduction

Chronic kidney disease (CKD) is the third most common cause of death after cancer and heart disease, and has become one of the major diseases that threaten global public health (1). China has the world's largest population of CKD patients, and the mortality rate among children with CKD is 30 times that among healthy children (2). Due to the long treatment cycle, complicated medication requirements, and many complications (metabolic disorders, infections and heart failure, etc.), caregivers are under great pressure (3), which has been shown to seriously affect their social interaction and quality of life (4). Studies have confirmed that good social interaction and minimal loneliness are associated with positive emotions (5–7), whereas less social support and greater loneliness are associated with insufficient self-demand and reduced social interaction (8, 9). At the same time, social support and loneliness are also associated with the occurrence, development, and mortality of physiological and psychological diseases, such as coronary heart disease and depression (10, 11).

Outbreaks of infectious disease causes the public to be anxious and to underestimate the possibility of their own survival (12, 13). Furthermore, coronavirus disease 2019 (COVID-19) poses a major threat to the global medical system (14, 15). Insufficient social support has significantly increased anxiety and stress among first-line medical staff in Wuhan (16); at the same time, the resulting loneliness and unemployment have exposed more children and women to domestic violence (17). During the outbreak of COVID-19, the management of children with CKD in China was fraught with challenges. The outbreak of infectious disease caused sharp reductions in social communication, shortages of living and pandemic prevention materials, a rising unemployment rate, and increasing family economic pressure. At the same time, due to the dysfunction of the social support system, isolation measures interrupted the traditional “face-to-face” diagnosis and treatment modes, resulting in delayed treatment of children. Evidence shows that caregivers of children with chronic diseases, such as tumors, have low social support and a high sense of loneliness (18–20), but there have been no studies evaluating the association between social support and loneliness in caregivers of children with CKD during the COVID-19 pandemic.

Therefore, we aimed to evaluate the social support and loneliness status of caregivers of children with CKD during the COVID-19 pandemic. This knowledge will allow us to develop suitable interventions to improve social support and loneliness among these caregivers in order to improve both the quality of life of caregivers and their patients.

## Materials and Methods

### Study Design

We conducted a cross-sectional study in two general pediatric hospitals in the western region of China during March 2020. The Hospital Information System was applied to collect data for children who were diagnosed with CKD between January 1, 2016 and November 31, 2019 and underwent general hospitalization. Then we invited the caregivers of children with CKD to recruit others caregivers for healthy children with a similar living environment and living standard through the “snowball method.”

### Subjects

We included caregivers who could use smartphones and were willing to give informed consent. We excluded caregivers who had a history of mental illness or other diseases that could impact their quality of life. For caregivers of children with CKD, we specifically recruited children with diagnosed CKD (21), and for caregivers of healthy children, the children currently had no diseases.

### Training of Investigators

Uniform training was provided for five investigators comprised of two nurses (professional titles: nurse or above; educational level: undergraduate or above) and three doctors (professional titles: attending physician or above; educational level: master degree or above). The training included: (1) research purpose and methods; (2) questionnaire types, evaluation points, and explanations of items; (3) methods and techniques for collection of questionnaires; and (4) interpretation and signature methods for the informed consent form.

### Investigation Method

In order to avoid person-to-person transmission during the COVID-19 epidemic, the researchers could only contact the subjects via telephone and the internet, so only verbal informed consent could be reached, and the study was approved by the Ethics Committee of Children's Hospital of Chongqing Medical University [2020(42)]. First, the researchers contacted the caregiver by telephone to explain the investigation's purpose, method, and possible risks, and to obtain verbal informed consent. Second, we sent the questionnaire to the caregivers online and guided them on how to complete it using electronic devices. Next, the quality of the questionnaire, including whether there were any missing items or errors, was checked by two investigators every day after the survey. Once missing items or errors were found, two investigators would call the participants for verification.

### Data Collection

We collected demographic data and COVID-19–related data as well as the scores on a social support scale and a loneliness scale. The collected demographic data included region, gender and age of children, rural or urban, caregiver-children relationship, caregiver occupation and education, and annual household income. The collected COVID-19–related data included whether relatives suffered from COVID-19, suspected virus exposure history, living status (whether segregated), fear of being infected with COVID-19, and worry about economic pressure.

### Social Support Rating Scale

The Social Support Rating Scale was used to measure the social support level in the current study. The scale includes subjective support (four items), objective support (three items), and utilization of support (three items). The scale has a total of 10 entries with a total score of 66 points. A higher score corresponds to a higher the level of social support. The Cronbach's α of the scale is 0.823 (22–24).

### Loneliness Scale

We used the Chinese version of the Loneliness Scale (ULS-8) to measure loneliness among the caregivers (25). There are eight entries in this scale, including six solitary positive-order entries and two non-solitary reverse-order entries. Each question is answered on a scale from 1–4, which correspond to never, rarely, sometimes, and always, respectively, and the total score of the scale is 32 points. A higher total score indicates a higher level of loneliness. The Cronbach's α for this scale in this study is 0.848 (26).

### Statistical Analysis

Logistic regression was used to establish the propensity scoring (PS) model including the following 13 variables: region, children's gender, age range, rural vs. urban, caregiver–children relationship, caregiver occupation/education, annual household income, whether relatives suffered from COVID-19, suspected virus exposure history, living status, worry about infection with COVID-19, and worry about economic pressure. The 1:1 nearest neighbor matching method was used with a caliper value set at 0.02 to PS match caregivers of children with CKD to caregivers of healthy children, and matching was restricted to within the common support region. We checked the balance of variables before and after matching by examining the absolute standardized differences between caregivers of CKD children and caregivers of healthy children, and a high quality of the matching was considered when the variables balance between the groups showed an absolute standardized difference <10% (27). After the PS match, paired *t*-tests were used to compare the differences in social support and loneliness outcomes. Pearson's test was used to assess the correlation between social support and loneliness. *P*-values < 0.05 were considered statistically significant. The SPSS 26.0 and R 4.0.3 statistical software programs were utilized to conduct all data analyses.

## Results

### Baseline Characteristics

**Figure 1:**
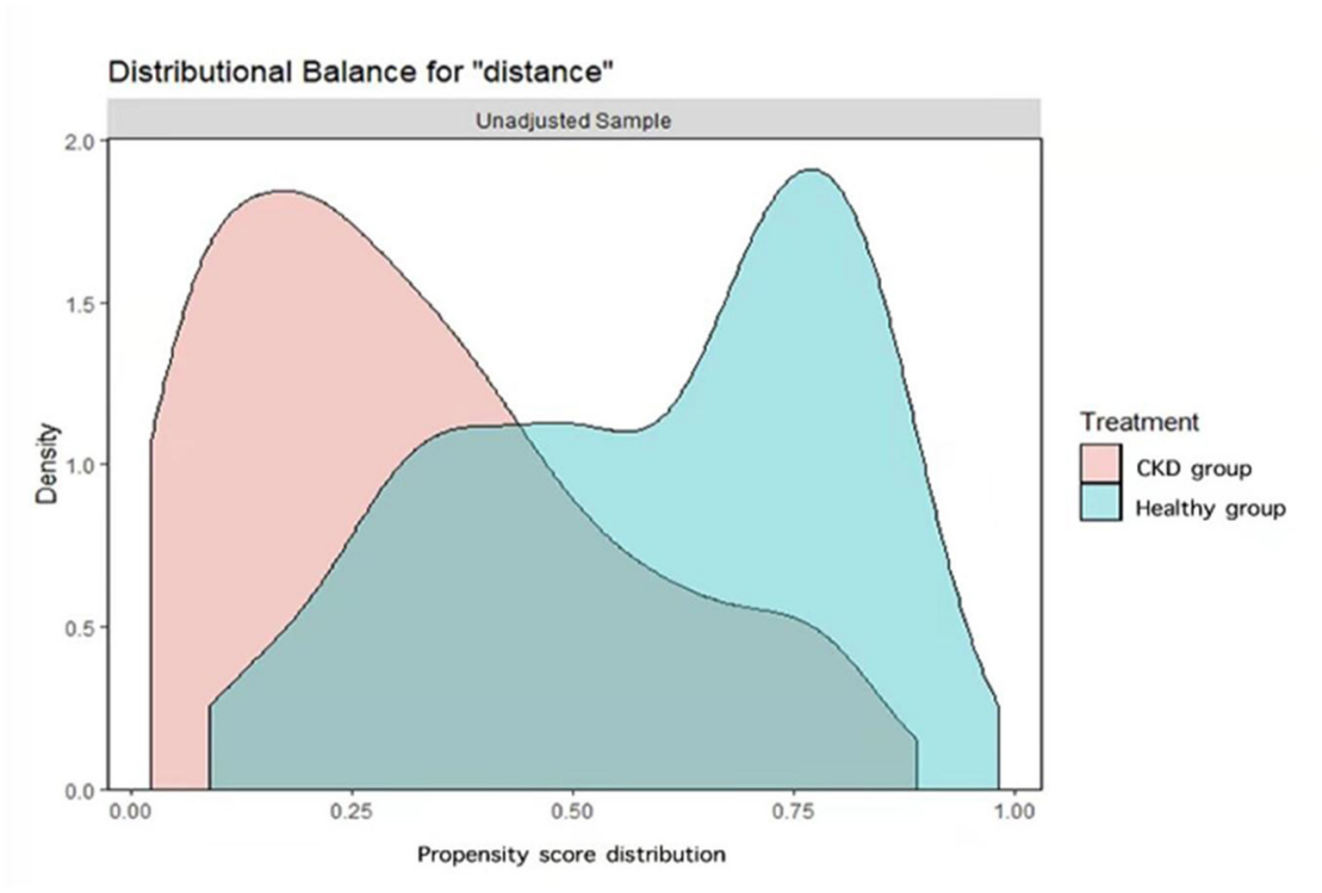
Overlay of Kernel density distributions of propensity scores for caregivers of children with CKD and caregivers of healthy children before propensity score matching.

**Figure 2:**
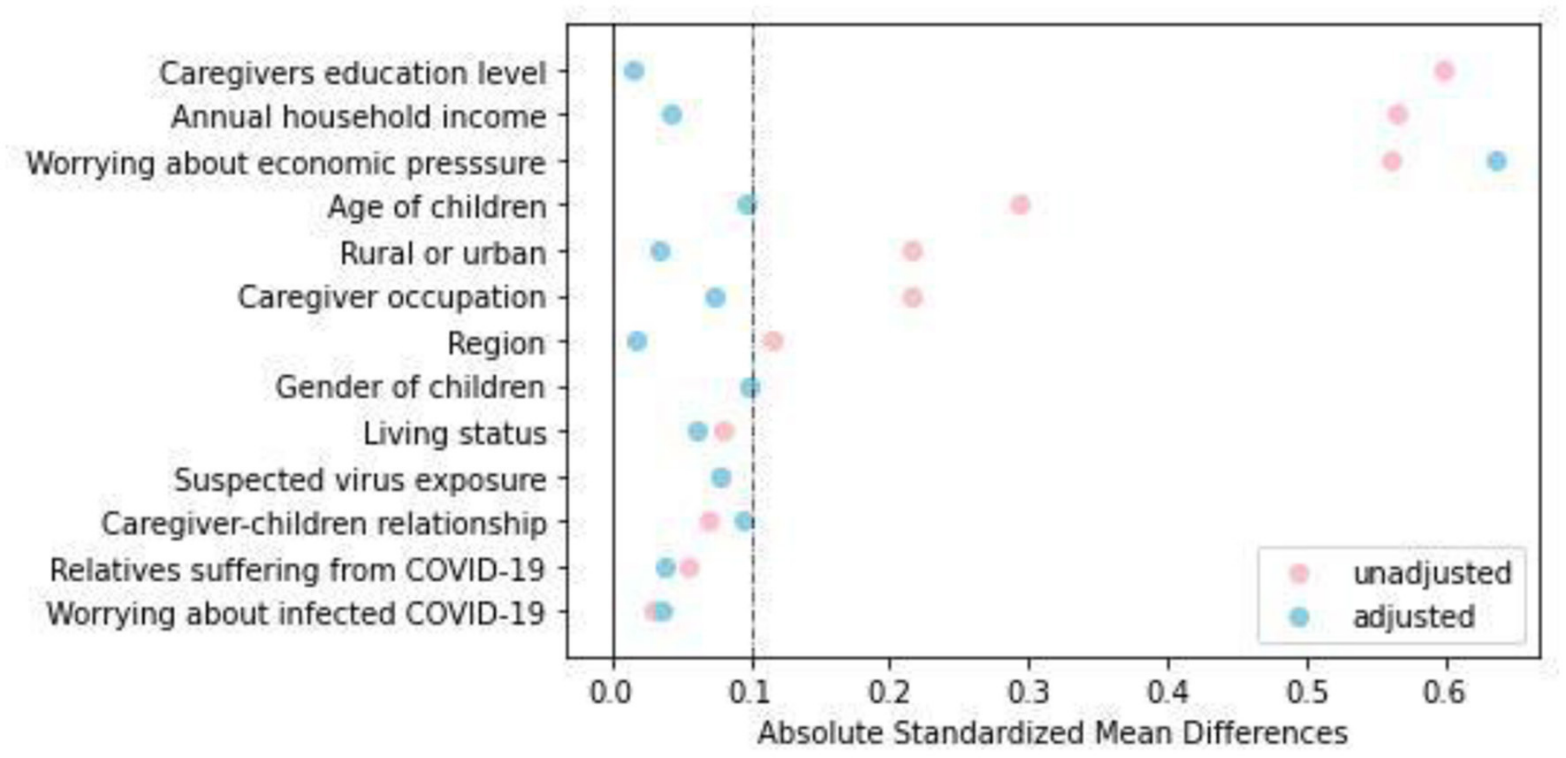
Dot plot of absolute standardized mean differences (Cohen's d) for all variables before and after matching.

A total of 562 caregivers from 13 provinces were recruited, including 247 caregivers of children with CKD and 315 caregivers of healthy children. After 1:1 matching, 202 caregivers were included in each group. As shown in Figure 1, before PSM, there was a common support area to perform PSM, and participants were predominately matched within the common region. Significant differences were observed between the two groups in terms of region [Standardized Mean Difference (SMD) = 0.117], age of children (SMD = 0.293), rural or urban (SMD = 0.215), caregiver occupation/education (SMD = 0.215 and 0.599, respectively), annual household income (SMD = 0.565), and worry about economic pressure (SMD = 0.562). After PSM, except for worrying about economic pressure (SMD = 0.636), the baseline characteristics of the two groups were comparable, as shown in Table 1 and Figure 2.

**Table 1:** Comparison of baseline data between the observation group and control group before and after PSM.

**Covariate**	**Before PSM**			**After PSM**		
	**CKD group** **(*n* = 247)**	**Healthy group (*n* = 315)**	**SMD**	**CKD group** **(*n* = 202)**	**Healthy group (*n* = 202)**	**SMD**
Region			0.117			0.017
Chongqing	101(40.9%)	145(46.9%)		84(41.6%)	84(41.6%)	
Sichuan	94(38.1%)	132(41.0%)		79(39.1%)	82(40.6%)	
others	52(21.1%)	38(12.1%)		39(19.3%)	36(17.8%)	
Gender of children (Boy)	96(38.9%)	146(46.3%)	0.100	82(40.6%)	96(47.5%)	0.098
Age of children (years)			0.293			0.096
<1	1(0.4%)	14(4.4%)		1(0.5%)	7(3.5%)	
1–3	11(4.5%)	34(10.8%)		10(5.0%)	14(6.9%)	
3–6	45(18.2%)	64(20.3%)		41(20.3%)	38(18.8%)	
6–12	114(46.2%)	114(36.2%)		87(43.1%)	76(37.6%)	
12–18	76(30.8%)	89(28.3%)		63(31.2%)	67(33.2%)	
Rural	116(47.0%)	102(32.4%)	0.215	95(47.0%)	100(49.5%)	0.035
Caregiver-children relationship			0.070			0.096
Mother	180(72.9%)	212(67.3%)		144(71.3%)	132(65.3%)	
Father	62(25.1%)	90(28.6%)		54(26.7%)	64(31.7%)	
Grandparents/Grandpa	5(2.0%)	13(4.1%)		4(2.0%)	6(3.0%)	
Caregiver occupation			0.215			0.074
Civil servant/institution	31(12.6%)	60(19.0%)		30(14.9%)	33(16.3%)	
Private company employees	41(16.6%)	83(26.4%)		37(18.3%)	41(20.3%)	
Self-employed	83(33.6%)	103(32.7%)		68(33.7%)	66(32.7%)	
Retirees	2(0.8%)	5(1.6%)		2(1.0%)	4(2.0%)	
Farmer	87(35.2%)	58(18.4%)		63(31.2%)	52(25.8%)	
Unemployed	3(1.2%)	6(1.9%)		2(1.0%)	6(2.9%)	
Caregivers' education level			0.599			0.015
Junior high school and below	127(51.4%)	86(27.3%)		86(42.6%)	79(39.1%)	
High school	60(24.3%)	94(29.8%)		56(27.7%)	70(34.7%)	
College (higher vocational education)	34(13.8%)	39(12.4%)		34(16.8%)	24(11.9%)	
Bachelor's degree and above	26(10.5%)	96(30.5%)		26(12.9%)	29(14.4%)	
Annual household income (×10,000 RMB)			0.565			0.043
<3	111(44.9%)	82(26.0%)		79(39.1%)	80(39.6%)	
3–8	88(35.6%)	105(33.3%)		75(37.1%)	78(38.6%)	
8–15	40(16.2%)	69(21.9%)		40(19.8%)	39(19.3%)	
>15	8(3.2%)	59(18.7%)		8(4.0%)	5(2.5%)	
Relatives suffering from COVID-19	7(2.8%)	3(1.0%)	0.055	6(3.0%)	3(1.5%)	0.039
Suspected virus exposure	9(3.6%)	4(1.3%)	0.077	8(4.0%)	2(1.0%)	0.077
Living status			0.079			0.062
Self-segregation	312(99.0%)	238(96.4%)		194(96.0%)	199(98.5%)	
Medical observation	3(1.0%)	9(3.6%)		8(4.0%)	3(1.5%)	
Worry about infected COVID-19	181(73.3%)	236(74.9%)	0.030	146(72.3%)	151(74.8%)	0.037
Worry about economic pressure	173(70.1%)	125(36.7%)	0.562	142(70.3%)	84(41.6%)	0.636

### Comparison of Social Support and Loneliness After PSM

As shown in Table 2, After PSM, the individual scores for social support (subjective support, objective support, utilization) among caregivers of healthy children are higher than those for caregivers of children with CKD (*P* < 0.002), and the loneliness score was higher for caregivers of children with CKD than for caregivers of healthy children (*P* = 0.008).

**Table 2:** Comparison of social support and loneliness scores between observation group and control group before and after PSM.

**Outcome indicators**	**Before PSM**			**After PSM**		
	**CKD group** **(*n* = 247)**	**Normal group (*n* = 315)**	**CKD group** **(*n* = 202)**	**Normal group** **(*n* = 202)**	***t***	***P***
Social support	36.0 ± 8.5	39.5 ± 7.2	35.7 ± 8.4	40.3 ± 7.0	4.653	<0.001
Subjective support	22.5 ± 4.8	23.8 ± 3.9	22.2 ± 4.9	24.0 ± 3.8	3.124	0.002
Objective support	6.7 ± 3.5	8.2 ± 3.5	6.6 ± 3.3	8.7 ± 3.5	4.374	<0.001
Utilization	6.8 ± 2.1	7.5 ± 2.1	6.9 ± 2.1	7.7 ± 2.0	3.359	0.001
Loneliness	16.8 ± 4.7	16.0 ± 4.7	16.8 ± 4.6	15.7 ± 4.6	−2.552	0.008

### Correlation Between Social Support and Loneliness Among Caregivers of Children With CKD After PSM

As shown in Table 3, correlation analysis showed that the total score for social support of the caregivers of children with CKD was positively correlated with each dimension of social support (subjective support, objective support and utilization; *r* = 0.693–0.906, *P* < 0.001) and negatively correlated with loneliness (*r* = −0.598, *P* < 0.001).

**Table 3:** Pearson correlation analysis of social support and loneliness among caregivers of CKD children after PSM (*n* = 202).

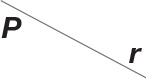	**Social support**	**Subjective support**	**Objective support**	**Utilization**	**Loneliness**
**Social support**	1	0.906	0.751	0.693	−0.598
		<0.001	<0.001	<0.001	<0.001
**Subjective support**		1	0.472	0.540	−0.579
			<0.001	<0.001	<0.001
**Objective support**			1	0.319	−0.347
				<0.001	<0.001
**Utilization**				1	−0.487
					<0.001
**Loneliness**					1

## Discussion

Our study showed that caregivers of children with CKD had less social support and greater loneliness than caregivers of healthy children. We also found that social support was negatively correlated with loneliness among caregivers of children with CKD. This is the first study to explore the current situation of the social support system and loneliness of caregivers of sick children during the COVID-19 pandemic.

Our results revealed that caregivers of children with CKD were more worried about the economy than were caregivers of heathy children. This finding is similar to that of Didsbury et al., which showed the economic status of families of children with CKD was lower (28). Studies have shown that the cost of treatment and care for CKD is huge (29), and coupled with the shutdown of economies during the COVID-19 pandemic, this further exacerbates the economic pressure on families with children with CKD and reduces their opportunities to receive financial assistance. More support should be given in terms of financial aid to help caregivers of children with CKD.

During the COVID-19 outbreak, 84.7% of Chinese residents were quarantined at home for 20–24 h a day, and one study reported that 75.2% of residents were worried about virus infection and 28.8% were anxious (30). Another study found that home quarantine was positively correlated with low levels of social ability and high levels of stress (31). In our study, caregivers of children with CKD had worse social support and greater loneliness than caregivers of healthy children. This may be related to the caregiver's fear of the epidemic of infectious disease, economic concerns, and worry about interruption of diagnosis and treatment of children as well as the pressure of disease care, as all of these feelings lead to a decline in the sense of social support and increased sense of loneliness. (1) The diagnosis and treatment of CKD is a long process, and caregivers of children with CKD are prone to social isolation (32, 33). (2) Children with CKD are managed in large specialized hospitals, and preventive measures of COVID-19 interrupted the transportation and information needed for long-term outpatient visits. The children's diagnosis and treatment were interrupted, and there was a reduction in objective social support due to the dysfunction of the social support system (34). (3) COVID-19 greatly threatens the life of immunocompromised children with CKD, likely making the entire family overly self-protective with greater social fears, further increasing the risk of loneliness.

Our study has some limitations. First, we cannot confirm the causality of the correlations determined. However, our findings provide us with an understanding of the association between social support and loneliness in this vulnerable group of caregivers. Second, we implemented our questionnaire using online platforms, and thus, we may have missed caregivers who are not technology literate. However, as our caregivers were largely young, the impact of missing this group of caregivers was minimal. Third, we did not use a randomized approach to conduct our sampling of caregivers of healthy children. Since highly motivated people usually choose to participate when convenience sampling is employed, the results may be exaggerated relative to the situation among the whole population. However, given that the pandemic was developing dynamically, we were constrained logistically. Last, this cross-sectional study can only investigate the current situation of caregivers of children with CKD during the COVID-19 epidemic; thus the causal relationship between the conclusion (social support and loneliness) and COVID-19 was not confirmed, and the influence of pre-COVID-19 baseline level cannot be excluded. Further study after COVID-19 epidemic is needed to rule out the confounding effects of the disease itself.

In conclusion, we found that caregivers of children with CKD had poorer social support and greater loneliness than caregivers of healthy children during the COVID-19 pandemic. We also found that social support was inversely correlated with loneliness. Potential interventions can be developed and implemented to support these caregivers financially and psychologically during the current pandemic and future pandemics to improve the quality of life of the caregivers and their children.

## Data Availability Statement

The raw data supporting the conclusions of this article will be made available by the authors, without undue reservation.

## Ethics Statement

The studies involving human participants were reviewed and approved by the Ethics Committee of Children's Hospital of Chongqing Medical University [2020(42)]. Informed consent to participate in this study was provided by the participants' legal guardian/next of kin.

## Author Contributions

XC and MW: research idea and study design. XL, HZ, HY, and DW: data acquisition/analysis/interpretation. DW, LS, and XL: resources. LS and DW: literature search. YZ and LS: statistical analysis. LS and HZ: writing–original draft. XC, HY and MW: supervision and mentorship. Each author contributed important intellectual content during manuscript drafting or revision, accepts personal accountability for the author's own contributions, and agrees to ensure that questions pertaining to the accuracy or integrity of any portion of the work are appropriately investigated and resolved.

## Conflict of Interest

The authors declare that the research was conducted in the absence of any commercial or financial relationships that could be construed as a potential conflict of interest.
